# Predicting in-hospital length of stay: a two-stage modeling approach to account for highly skewed data

**DOI:** 10.1186/s12911-022-01855-0

**Published:** 2022-04-24

**Authors:** Zhenhui Xu, Congwen Zhao, Charles D. Scales, Ricardo Henao, Benjamin A. Goldstein

**Affiliations:** 1 Department of Biostatistics and Bioinformatics, Duke University, 2424 Erwin Road, Suite 1104, Durham, NC 27705 USA; 2 Duke Clinical Research Institute, Duke University, Durham, NC USA; 3 Department of Population Health Sciences, Duke University, Durham, NC USA; 4 Department of Surgery, Duke University, Durham, NC USA; 5 Department of Electrical and Computer Engineering, Duke University, Durham, NC USA

**Keywords:** Electronic health records, Machine learning, Clinical decision support, Surgical outcomes

## Abstract

**Background:**

In the early stages of the COVID-19 pandemic our institution was interested in forecasting how long surgical patients receiving elective procedures would spend in the hospital. Initial examination of our models indicated that, due to the skewed nature of the length of stay, accurate prediction was challenging and we instead opted for a simpler classification model. In this work we perform a deeper examination of predicting in-hospital length of stay.

**Methods:**

We used electronic health record data on length of stay from 42,209 elective surgeries. We compare different loss-functions (mean squared error, mean absolute error, mean relative error), algorithms (LASSO, Random Forests, multilayer perceptron) and data transformations (log and truncation). We also assess the performance of two stage hybrid classification-regression approach.

**Results:**

Our results show that while it is possible to accurately predict short length of stays, predicting longer length of stay is extremely challenging. As such, we opt for a two-stage model that first classifies patients into long versus short length of stays and then a second stage that fits a regresssor among those predicted to have a short length of stay.

**Discussion:**

The results indicate both the challenges and considerations necessary to applying machine-learning methods to skewed outcomes.

**Conclusions:**

Two-stage models allow those developing clinical decision support tools to explicitly acknowledge where they can and cannot make accurate predictions.

**Supplementary Information:**

The online version contains supplementary material available at 10.1186/s12911-022-01855-0.

## Background

At the beginning of the COVID-19 pandemic, surgical leadership was tasked with determining which elective surgeries would necessitate the usage of additional resources, with the intention of potentially delaying them. In response, we developed and implemented a clinical decision support (CDS) tool to predict anticipated length of stay (LOS), need for intensive care unit, need for mechanical ventilation and need to be discharged to a skilled nursing facility [1]. Overall, the model had clinically meaningful predictive performance (high sensitivity of the high-risk group and high negative predictive value of the low-risk group) and has been used by our operations team to make scheduling decisions when hospital resources became strained during various waves of the pandemic.

Initially, we had intended to predict hospital LOS as a continuous outcome. However, internal testing yielded a poor performing model. Given the need to quickly implement a CDS tool we instead categorized LOS into 4 categories (0–2 days, 2–4 days, 4–7 days and 7 + days) and treated it as a classification task (Additional file 1: Tables S1 show the classification results on the test data). These cut-points were subjectively chosen, based on guidance from the clinicians that would be using the CDS. Our treatment of LOS is not unique, as many other studies have modeled LOS as a categorical variable [2–7]. While most statistical learning algorithms can be equally applied to classification and regression tasks, the right skewness (i.e. long tail) of LOS makes it challenging to model. Methods that have been applied to right-skewed data include truncation or log transformation [8] or the application of non-parametric machine learning methods [9]. Other modelling approaches also include time-to-event based Cox models [10] and discrete time logistic regression models [11]. However as explored below, these approaches do not always achieve ideal performance.

Predicting LOS as a continuous outcome has the advantage of being able to provide the end user of a CDS with a more precise estimate of the outcome. As such, in this paper, we systematically consider different options for predicting in-hospital LOS after an elective surgery. Since we had to quickly implement a model in response to the COVID-19 pandemic, we were not able to consider more subtle questions of optimal modelling strategy. While the original model had good classification—particularly for the extreme long and short LOSs (Additional file 1: table S1)—we wanted to see how best to develop a model that predicted LOS as a continuous outcome. Ultimately we approach modeling LOS as a two-stage process, first separating the majority of patients with a short LOS from the minority of patients with a long LOS. Then we seek to predict the continuous response for the majority with a short LOS, tacitly acknowledging that accurately predicting the long LOS is not possible. A two-stage model is commonly applied to skewed outcomes in the health-economic area where the response variables is a combination of excessive zeros and positively skewed distribution. Smith et al. [12] used simulation studies to show that a two-stage approach can produce results that are more robust. We tailored this idea to the clinical setting, where we strike a balance between discrimination of prolonged LOS and precise prediction of majority of population. In other settings, we [13], and others [14], have used a two-stage models to predict skewed outcomes arising from zero-inflated problems in which excessive zeros are first modelled by a classifier and then positive values are modelled by a regressor. In our study, there is also excessive short LOSs resulting in imbalanced data as excessive zeros in the zero-inflation problem. We first identified short LOSs and modelled those samples by a regressor. We detail the impact of different considerations such as loss-function, algorithm, data transformations, and data set-up. We ultimately conclude—based on our data—that a two-stage model that first separates out long stays from shorts stays and then tries to predict only on short stays has the most practical real-world performance.

## Materials and methods

### Setting

We abstracted data from the Duke University Health System (DUHS) electronic health record (EHR) system. DUHS consists of three hospitals—1 tertiary care center and 2 community hospitals—and has had an integrated EPIC EHR system since 2014.

### Data

#### Case definition

As described previously [1], we abstracted information on all elective inpatient procedures performed at a DUHS hospital from January 1 2017 to March 1 2020. While there is no formal definition of an elective procedure, we included all procedures that had a designation of “Surgery Admit Inpatient.” This is an indication that the patient was admitted for the purposes of surgery and not via, for example, the emergency department. We included both adult and pediatric procedures.

#### Definition of predictors

The intent of the CDS tool was to make predictions the week prior to when the case was scheduled. As such, we abstracted patient and procedure specific information known prior to the procedure. This included demographic characteristics, procedure CPT codes, service line, medication history, comorbidities and service utilization history. This resulted in a total of 44 unique predictor variables. (See Additional file 1: Table S2).

### Analytic approach

We first describe the analytic data. We then took a systematic approach to considering different options for modeling LOS as outlined in Table 1. To do so, we first divided the data randomly into training (2/3) and testing (1/3) sets. We used fivefold cross-validation on the training data to optimize each model’s performance and compare the overall performance of each model. After choosing the best modeling approach, we applied it to the held out testing set. We used bootstrap resampling to estimate 95% confidence intervals for the final estimates.

**Table 1:** Overall analytic approach

Algorithm choice	Loss function	Data manipulation	Modeling approach
LASSO	Mean squared error (MSE)	Original data	One-stage approach
Random forest	Mean absolute error (MAE)	Log data	Two-stage approach
Multilayer perceptron	Mean relative error (MRE)	Truncated data	

This table guides the analytic approach in this study. We compared different algorithm choices, loss functions, data manipulations and modeling approaches

#### Algorithm choice

We first considered the performance of three different algorithms: LASSO regression [15], Random Forest (RF) [16], Multi-Layer Perceptron (MLP). Each approach has their own relative strengths and weaknesses when considering skewed data. LASSO is a form of linear regression that controls overfitting by penalizing the sum of the norm of the regression coefficients. While a powerful algorithm, it can be susceptible to outlier outcome values and may require specific transformations (e.g. log transformation) to satisfy linear assumption. It also ignores interaction terms unless manually added. In comparison, non-parametric methods do not make distributional assumptions nor require transformation of outcomes and predictors. RF is an ensemble tree method that is less influenced by outliers. However, this can also make modeling such tails more challenging. Finally, an MLP is a deep-learning, neural network, model that can capture complex relationships. However, they also require much more data than LASSO and RF due to the larger number of parameters and can become inconsistent or unstable given different initial status [17]. While non-parametric methods offer more flexibility with fewer assumptions, producing strong results [18, 19], regression models can also produce reliable results given appropriate transformation of outcomes [20] and tend to do better for extrapolating to testing samples beyond the range of training samples [21]. We used fivefold internal cross-validation to optimize the tuning parameters of each algorithm.

#### Loss function

While mean squared error (MSE) is the most commonly used loss function for continuous outcomes, when the data are skewed, loss functions can have different interpretations and performance. As such, we considered two additional loss functions: mean absolute error (MAE) and mean relative error (MRE). These loss functions are defined as follows:

$$MSE=\frac{\sum_{i=1}^{n}{\left({\widehat{y}}_{i}-{y}_{i}\right)}^{2}}{n}$$

$$MAE=\frac{\sum_{i=1}^{n}|{\widehat{y}}_{i}-{y}_{i}|}{n}$$

$$MRE=\frac{\sum_{i=1}^{n}\frac{|{\widehat{y}}_{i}-{y}_{i}|}{{y}_{i}}}{n}$$

where n is the number of samples and ${\widehat{y}}_{i}$ is the predicted LOS of the $i$ th observation and ${y}_{i}$ is the actual LOS of the $i$ th observation. One primary drawback of MSE with skewed data is that it tends to be more influenced by errors from extreme values. Conversely, MAE does not suffer from this. Moreover, the clinical interpretation of MAE is the most straightforward, that is the average deviation from the true LOS. MRE is a less commonly used loss function. It represents the proportion of prediction errors compared to the true values and in contrast to MSE is more likely to be influenced by smaller values.

We note that these loss functions were not designed to optimize the individual algorithms, but to guide our overall modeling process. These loss functions estimate error across the domain of the outcome while we ultimately focused on those with short LOSs (0–7 days). Thus, we designed a customized loss function to evaluate the two-stage model as a whole (see details in Customized Loss Functions).

#### Data manipulation

As others have shown [8], performing transformations of the outcome can improve modeling performance. Taking the log of a right skewed outcome can produce a more symmetric distribution [22], while truncating the outcome to remove outliers can alleviate the influence of extreme values [23]. In a regression context a log transformation is similar to modelling LOS via a Poisson or negative binomial regression model which others have done [8, 24]. We assessed both using a log transformation as well as a truncation of the outcome in the training data and compared the model performance given untransformed, truncated, log-transformed data. We used a truncation threshold set to 7 days, i.e., LOS > 7 days were reduced to be 7 days in the training set while the outcome values in the testing set still remained the same. The base of log transformation in our study was the natural log.

#### Two-stage approach

Finally, we assessed a two-stage approach for modeling LOS. In the first stage we constructed a classifier to predict a patient would have a short or long LOS (defined as >  = 7 days). The decision rule of identifying prolonged LOS was based on the obtaining a sensitivity of 15% for prolonged LOS on the training dataset. We chose this threshold based on inspection of the precision-recall curve (See Fig. 7). Next, among those with a predicted short LOS we fit an RF regressor to predict actual LOS. We again considered the impact of different data constructions for performing this two-stage model.

**Fig. 1:**
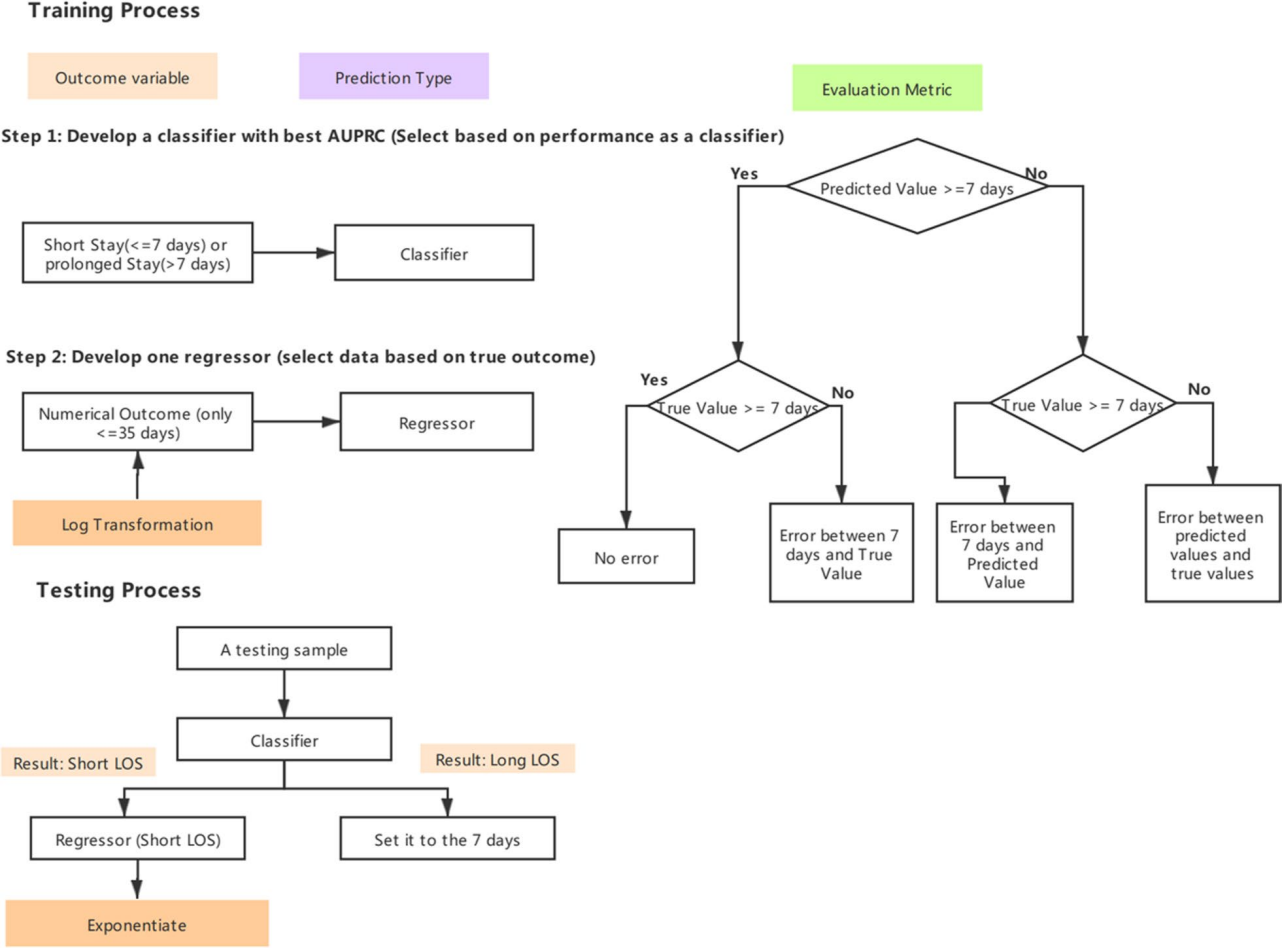
Flow chart of the two-stage model

We show the modeling process for the two-stage model in Fig. 1. In stage 1, we used all of the training samples to train the classifier. In stage 2 we used only the training samples with a LOS <  = 35 days to train the regressor. The threshold of the regressor was set higher than the threshold of the classifier to expose the regressor more frequently to rare cases. The participants with extremely prolonged LOS (LOS > 35 days) were not included in the regressor. To generate a new prediction in the test data, the classifier first classified all of the testing samples and only those classified as short LOS were fed into the regressor. The regressor then made continuous predictions to those labeled as short LOS.

#### Customized loss function

In order to evaluate the two-stage model we introduce a truncated loss function based on a hinge loss. We ascribe no loss if both predictions and true outcomes were larger than the threshold of the classifier (i.e. 7 days). Otherwise, we applied the MAE. If the true outcome >  = 7 days but the prediction < 7 days, we only measured the difference between the prediction and the threshold of the classifier.

All analyses were performed through Python 3.7. This work was determined exempt by our institution’s IRB.

## Results

We identified 42,209 elective procedure performed at DUHS hospitals from January 1, 2017 to March 1, 2020. Table 2 has basic descriptions based on LOS. There were demographic differences among those with longer and shorter stays indicating that the input variables should be useful for generating predictions.

**Table 2:** Descriptive statistics of predictors by LOS*

	0–2 days (n = 15,696)	2–4 days (n = 15,122)	4–7 days (n = 7226)	> = 7 days (n = 4165)
Demographics				
Age, years (mean, SD)	58.09 (17.59)	57.12 (19.10)	57.07 (20.19)	57.40 (21.44)
Sex = female (n, %)	7792 (49.6%)	8839 (58.5%)	3820 (52.9%)	1873 (45.0%)
Race (n, %)				
NHW**	12,020 (76.6%)	10,646 (70.4%)	5173 (71.6%)	2935 (70.5%)
NHB***	2567 (16.4%)	3364 (22.2%)	1477 (20.4%)	869 (20.9%)
Hispanic	342 (2.2%)	371 (2.5%)	181 (2.5%)	99 (2.4%)
Other	767 (4.9%)	741 (4.9%)	395 (5.5%)	262 (6.3%)
Smoke status = Ever (n,%)	4887 (31.1%)	4893 (32.4%)	2707 (37.5%)	1741 (41.8%)
BMI (n, %)				
Underweight	478 (3.0%)	605 (4.0%)	389 (5.4%)	311 (7.5%)
Normal	3248 (20.7%)	3235 (21.4%)	1767 (24.5%)	1108 (26.6%)
Overweight	5076 (32.3%)	4319 (28.6)	2167 (30.0%)	1253 (30.1%)
Obese	6866 (43.7%)	6950 (46.0%)	2895 (40.1%)	1490 (35.8%)
Service utilizations				
Hospital encounter counts (mean, SD)	0.24 (0.74)	0.26 (0.73)	0.36 (0.93)	0.55 (1.13)
Ambulatory encounter counts (mean, SD)	15.76 (17.12)	16.77 (18.29)	17.99 (19.45)	20.67 (22.90)
Emergency encounter counts (mean, SD)	0.16 (0.83)	0.20 (0.83)	0.23 (0.86)	0.32 (2.20)

*Procedure data, medicine history and comorbidities are not included in this table

**NHW: Non-Hispanic Whites

***NHB: Non-Hispanic Blacks

**Fig. 2:**
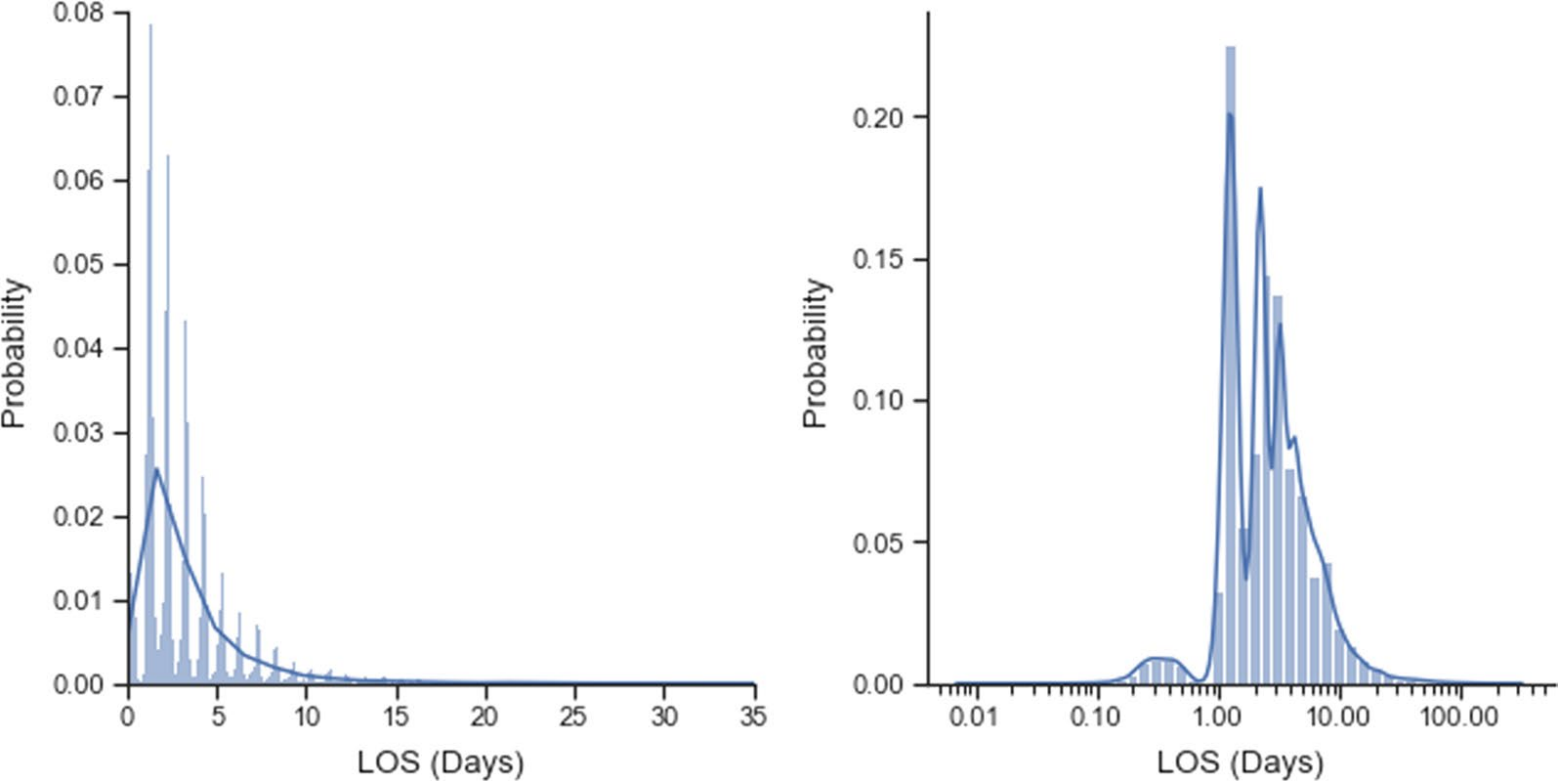
Histogram of LOS in Days (X-axis of left in original scale and right in logarithmic scale) Note the x-axis of the left hand side is truncated to 35 days

The distribution of the LOSs is shown in Fig. 2. As expected LOS is highly right skewed with the majority of patients has 0–4 days of LOS and 9.9% patients having a LOS >  = 7. The longest LOS was 323.35 days.

### Algorithm comparison

We compared the performance of LASSO regression, RF, and MLP algorithm. We then compared the best models selected from each algorithm through CV results on the training set, shown in Table 3. Across all loss functions, RF had the best performance, and we chose it as the algorithm to use going forward.

**Table 3:** Comparison of model performance between lasso, random forest and multilayer perceptron

	Lasso	Random forest	Multilayer perceptron
CV-MSE	22.924	**21.185**	24.370
CV-MAE	1.958	**1.877**	2.305
CV-MRE	1.006	**0.972**	1.036

Bolded values indicate minimized loss

### Loss functions

**Fig. 3:**
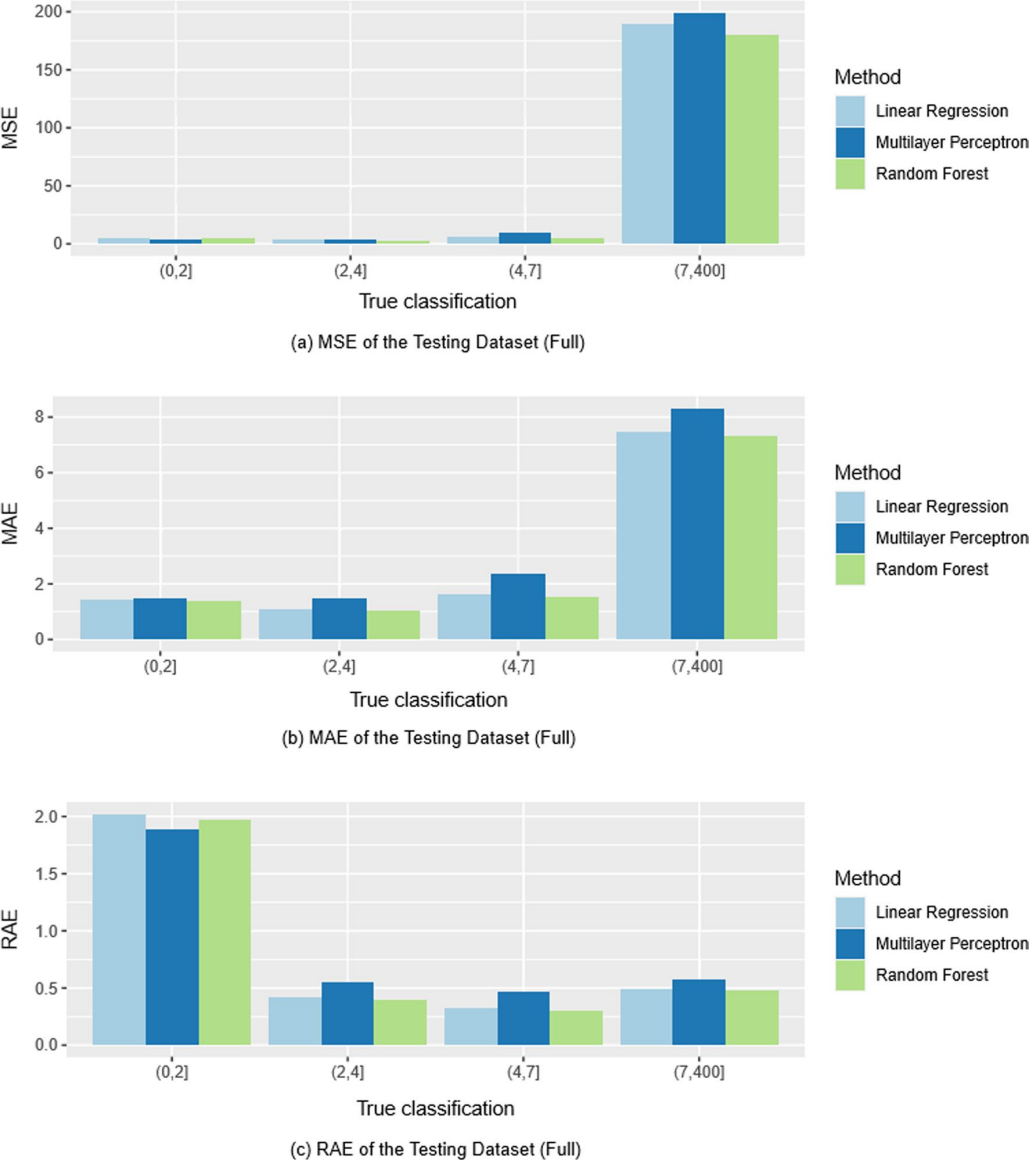
Stratified evaluation metrics of the full model

We further explored the impact of using different loss functions by grouping the evaluation metrics into bins for people with LOSs of 0–2, 2–4, 4–7 and >  = 7 days, respectively (Fig. 3). This highlights how each evaluation metric focuses on different clinical representations. For example, MSE has a greater loss on larger LOSs while MRE has greater loss on the smaller LOSs. Thus, if we choose to select our procedures based on MSE, our procedures will try to perform the best for extreme values (> = 7 days). Conversely, when evaluating based on MRE, our procedures seek to perform best on shorter LOSs (0–2 days).

After consultation with clinical collaborators, we ultimately decided that MAE was the best selection metric to use. We chose it because (1) it has the most straightforward clinical interpretation as the absolute difference between prediction and true values in days, and (2) MAE more evenly assigns weight on longer LOSs values (compared to MSE) while still placing greater weight on the longer LOSs (compared to MRE).

### Data set-up

**Fig. 4:**
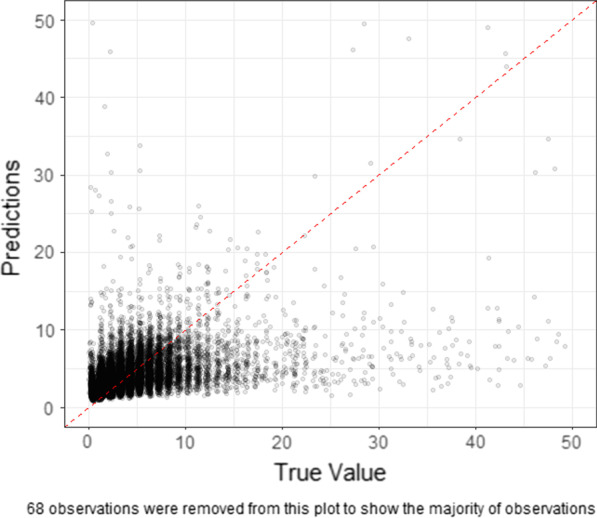
Predictions versus true values of untransformed data (RF)

Figure 4 shows a comparison of the predicted versus observed values from the RF model. It is clear that the longer LOSs are under-predicted. In particular, it was very hard for our model to make predictions greater than 14 days (which account for only 0.62% of all encounters).

We assessed the impact of a log transformation and a truncation at 7 days. Table 4 presents the models’ MAE and calibration as well as sensitivity for predicting >  = 7 days. The log and truncated models did not meaningfully improve modeling performance. In particular, the truncated model had a sensitivity of 0 since the model was not exposed to observations with LOS >  = 7 days. The lack of ability to predict patients with prolonged LOS might cause underestimation of overall hospital utilization.

**Table 4:** Comparison between untransformed, log, truncated, and two-stage outcome

	Untransformed LOS	Log LOS	Truncated LOS	Two-stage model
Customized loss function*	1.338	1.126	1.183	1.118
MAE	1.880	1.695	1.796	1.730
Calibration	0.528	0.429	0.317	0.418
Sensitivity < 7 days	0.970	0.990	1.00	0.990

*This is the two-stage loss function described in the methods section

### Two-stage model

Finally, we considered a two-stage modeling approach where we first generated a classifier to discriminate long from short LOS (stage 1) and then a regressor to predict LOS as a continuous variable among those with a short LOS (stage 2). We set LOS > 7 days as prolonged LOS and LOS < 7 days as short LOS. This threshold was based on empirical examination of the modeling results where most models have trouble predicting LOS of greater than 7 days (Fig. 4) and based on consultation with clinical collaborators.

We set the decision rule of the classifier by the sensitivity of prolonged and short LOSs. Based on the consistency with the one-stage model, we set this threshold to 99%. To make continuous predictions for the majority of observations, we selected a threshold with sensitivity of short LOS = 0.99 based on the fivefold internal CV on the training dataset.

**Fig. 5:**
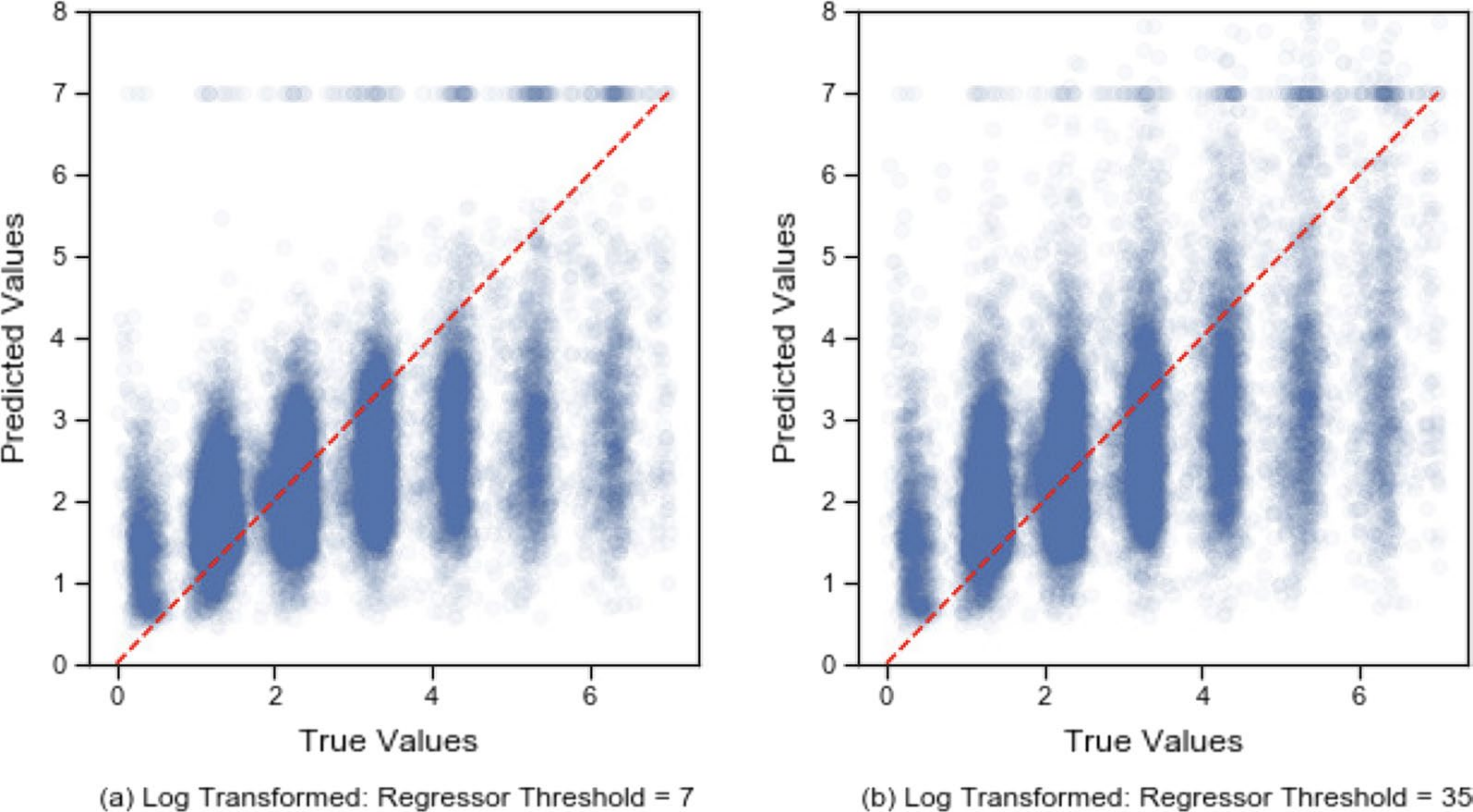
The comparison between different thresholds of the regressor and untransformed and log transformed LOS

To allow the model to predict beyond 7 days, we used training data within the regressor higher than the threshold of the classifier. Here, we referred the threshold of the regressor to the upper boundary of the training data fed into the regressor. The increased threshold of the regressor can also expose the model to more samples of rare cases. This method improves the model performance for those with 5–7 days LOS (Fig. 5) which results in decrease of MAE (Table 5).

**Table 5:** Different thresholds for the classifier and the regressor in the two-stage model

	Untransformed LOS			Log LOS		
	7	21	35	7	21	35
MAE	1.150	1.202	1.242	1.175	1.119	1.118
Calibration	0.339	0.454	0.476	0.320	0.408	0.418
Sensitivity < 7 days	0.991	0.984	0.978	0.991	0.989	0.988

Besides the decrease in MAE, increasing the threshold when training the regressor also has an advantage of improving sensitivity and calibration slope (Table 5). If the thresholds of the regressor and the classifier were equal, the model would underestimate higher values. The increased threshold adds more variability to the data while the majority predictions remain accurate. The final regressor threshold was set to 35 days.

**Fig. 6:**
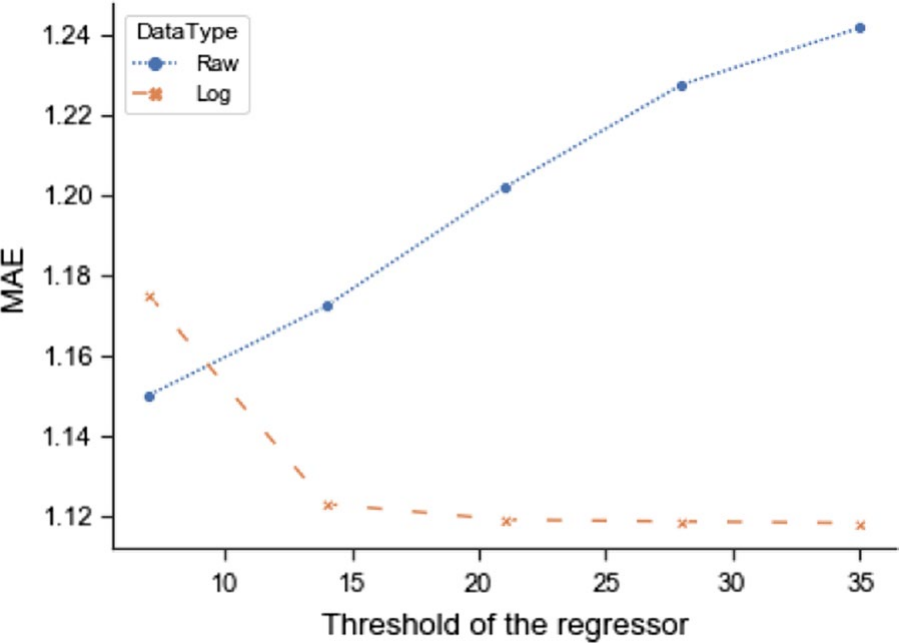
Truncated evaluation metrics of different regressor thresholds

One thing we noted was that using the natural data tended to overestimate the lower values. Log transformation eliminates this problem to some degree since log transformation enlarges the difference between lower values and shrinks the difference between large values. Figure 6 shows the trend of truncated MAE as the threshold of regressor increases. Although the MAE of untransformed data increases as the threshold increases, MAE of log transformed outcome decreases. Thus, log transformation has the advantage eliminating the adverse effects of introducing more extreme values.

### Results on the testing data

**Fig. 7:**
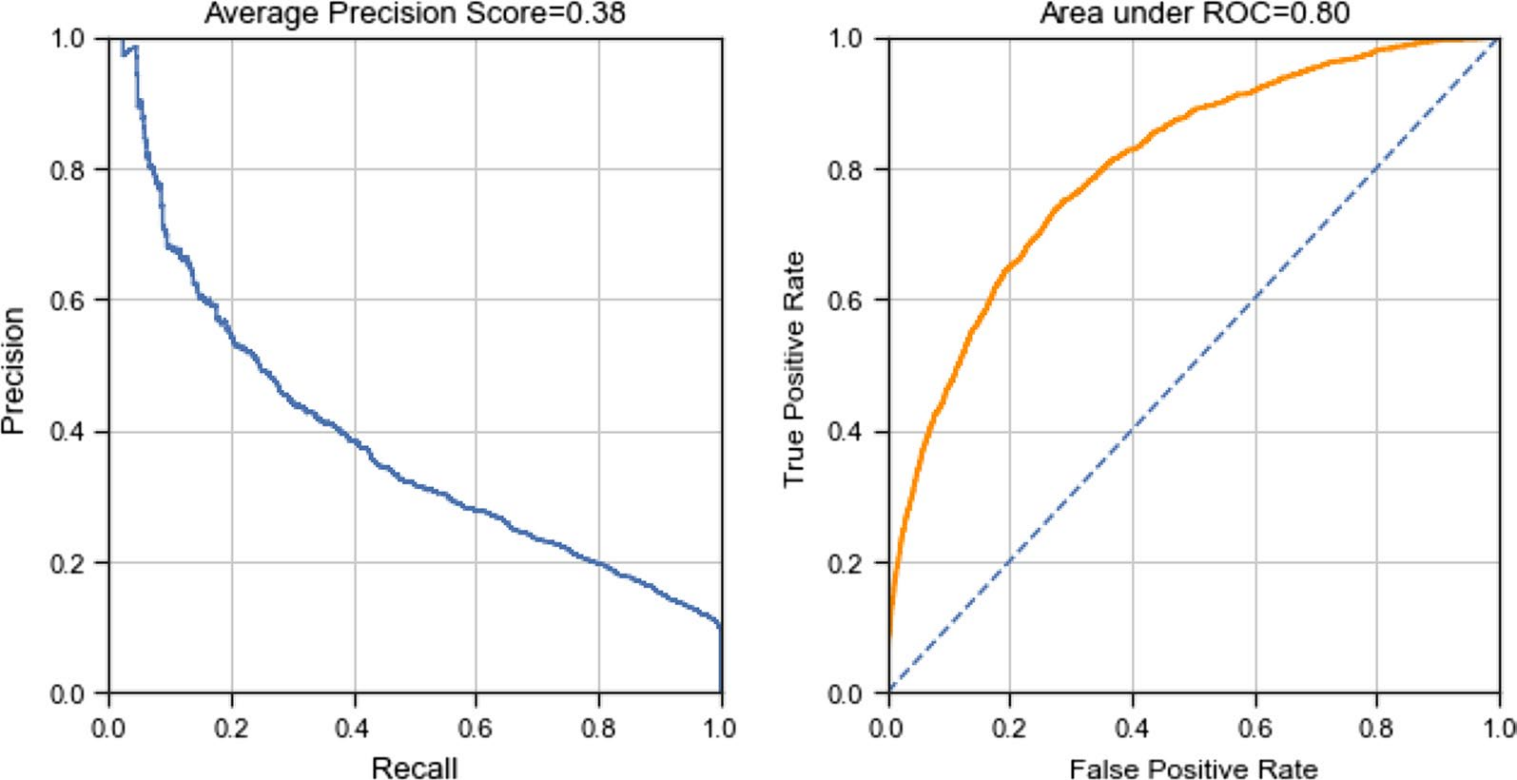
Precision-recall curve (average precision = 0.38) and receiving operating characteristics (AUC = 0.80) of the classifier (stage 1) on the testing dataset

Based on the tests performed on the training data we concluded that the best performing model is a two-stage model, using RF and log transforming the outcome on the second stage. We used the one-third held-out dataset to evaluate the performance of this model. The average precision (AP) of the classifier is 0.38 (Fig. 7).

**Fig. 8:**
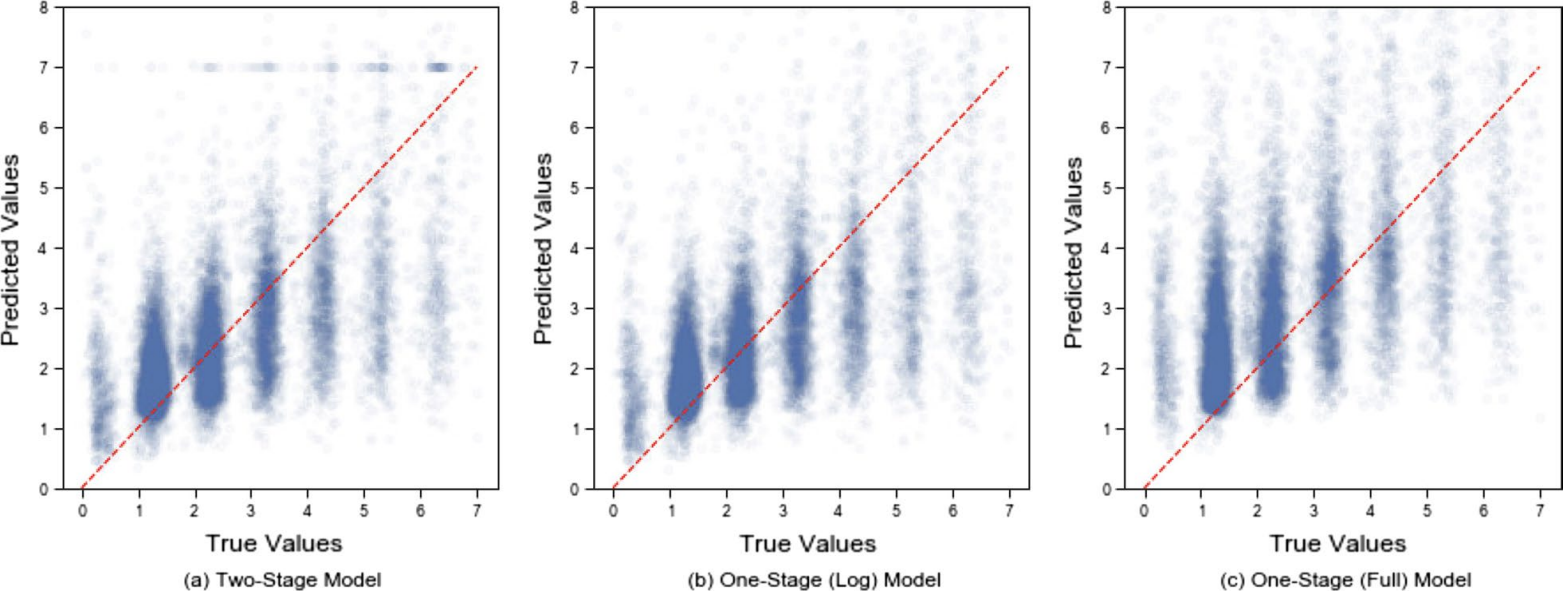
Predictions versus true values on the testing dataset

The truncated MAE is 1.1 on the testing dataset, indicating there is 1.1 days error of LOS prediction on average. The calibration slope is 0.44 indicating that there is still some under-prediction of LOS. This is primarily due to LOS > 4 days (Fig. 8).

The truncated MAE for those with 4–7 days LOS is 1.76 days while the MAE for those with 0–4 days is less than 1 day (Table 6). Our two-stage model performs slightly better than the one-stage model with log data. Log transformation improves the model performance significantly on the majority of data (0–4 days) while it performs worse on longer LOSs (> 4 days). The two-stage model compensates for the worse performance on LOS > 4 days to some degree. Patients with a larger true LOS are more likely to be misclassified as prolonged LOS (shown as horizontal blue dotted line in Fig. 8). Our model can predict patients with 0–4 days well while it tends to underestimate the LOS > 4 days.

**Table 6:** Stratified customized loss functions (MAE) of one-stage and two-stage models with 95% bootstrap confidence intervals

	One-stage model (log data)	Two-stage model (log data)	LASSO	RF	MLP
0–2 days	0.744 (0.739, 0.749)	0.736 (0.731, 0.741)	1.596 (1.585, 1.607)	1.313 (1.305, 1.321)	1.638 (1.626, 1.648)
2–4 days	0.713 (0.709, 0.718)	0.705 (0.700, 0.709)	1.046 (1.037, 1.058)	1.035 (1.027, 1.047)	1.155 (1.142, 1.168)
4–7 days	1.782 (1.771, 1.794)	1.760 (1.750, 1.772)	1.492 (1.474, 1.509)	1.586 (1.571, 1.600)	1.730 (1.713, 1.746)
0–7 days	0.927 (0.922, 0.930)	0.915 (0.911, 0.919)	1.358 (1.351, 1.366)	1.254 (1.248, 1.261)	1.464 (1.456, 1.471)

Finally, we tested the performance of the two-stage model during the COVID-19 period. While the LOSs stayed nearly identical (pre-March 2020: 2.30 [1.32, 4.18], post-March 2020: 2.29 [1.30, 4.18]) overall model performance was meaningful worse during the COVID-19, particularly for shorter LOSs (Table 7).

**Table 7:** Performance of two-stage model during COVID-19 period

	Two-stage model (01/01/17–03/01/20)	Two-stage model (03/01/20–02/22/22)
0–2 days	0.736 (0.731, 0.741)	2.298 (2.280, 2.313)
2–4 days	0.705 (0.700, 0.709)	1.251 (1.236, 1.263)
4–7 days	1.760 (1.750, 1.772)	2.324 (2.315, 2.333)
0–7 days	0.915 (0.911, 0.919)	1.934 (1.922, 1.944)

## Discussion

In this paper, we explored the challenge of developing a predictive model for a highly skewed outcome, LOS. When we first developed our clinical decision support tool, we decided to change LOS into a categorical outcome because we were not able to derive a satisfactory prediction when treating it as a continuous outcome [1]. However, discretizing a variable leads to loss of information and is generally not recommended [25]. Based on our empirical study, we found that the best approach was a hybrid two-stage approach that first uses a classifier to identify shorter LOSs and then uses a regressor to more finely predict the actual LOS. While the classification model we originally implemented had reasonably good performance—particularly for the shortest and longest LOSs—the continuous model adds additional predictive specificity for shorter LOS less than 7 days. Specifically, our final MAE suggests that our models predictions are off by less than 1 day (~ 16 h) for LOS < 4 days and less than 2 days for LOS between 4 and 7 days.

This work highlights some of the challenges with predicting LOS. There are many analytic choices one has to make when modeling such as outcome: including algorithm type, loss function, and variable transformations (see Table 1). Each of these choices had impacts on the final model. In our analysis, we concluded that RF performed best. While there is no universally best algorithm, in our setting, RF strikes a balance between being non-parametric (compared to LASSO) and less data demanding (compared to MLP).

We decided to use a MAE as our evaluation loss function. While MSE is most commonly used for continuous outcomes, it is recognized [24] that it is not appropriate for skewed outcomes since it places too much weight on the tails. We considered MRE, however note that it placed most of its weight on the shorter LOSs, inappropriate for our use case.

Finally, we assessed the impact of transformations of the outcome by log and truncation. While others have had success with such transformations [8, 9, 26], they did not perform as well in our data. Interestingly, the log transformation was preferable within the context of the two-stage model suggesting that such a transformation is only useful when the skew is minimal.

Ultimately, we concluded that we could not create a single continuous prediction model, settling on a two-stage model. While such models are typically used in zero-inflated problems [12, 14], we applied the two-stage model to identify a space where we could make finer predictions and where we could not. In particular, we concluded that we could predict LOSs less than 7 days accurately but could not predict those longer than that. While the improvement in MAE from the one to two stage model is statistically different, the difference is not very clinically meaningful. Since long LOSs are relatively rare, the miss-predictions do not overly affect the estimation of MAE. Based on reported work by others, it is likely that others would similarly benefit from a two-stage approach. Liu et al. [8] developed a series of regression models for LOS, reporting a MSE of 29,000, with only 55% of predictions being within 48 h of the actual LOS. Similarly, Verburg et al [24]. reported MAEs of no-better than 3 days for predicting ICU LOS. By implementing a one-stage model, we believe that we would be misleading clinical users. Instead, by choosing a two-stage model we are acknowledging that we cannot make accurate predictions for the longer LOSs. Ultimately, we believe that doing this ultimately helps to engender more trust in a CDS tool.

Given the nature of our predictor data, which consisted of pre-surgical information, it is not surprising that it is harder to predict longer LOSs. It is likely that if someone has a longer LOS that is going to be due to post-surgical complications that may not be predictable based on pre-procedure information. It is also possible that the sample size of patients with prolonged LOS will not be large enough to capture the characteristics of this subgroup of patients. Essentially, one can think of the classifier (i.e., the first-stage of the two-stage model), as first predicting likelihood for surgical complications. If there is low likelihood, we predict LOS, if there is high likelihood we acknowledge we cannot do any better given the information we have. Kumar et al.^27^ developed a two-stage model that first predicted LOS before admission and then utilized predictors 5 days after admission. The predictors after admission improved the predictive accuracy of prolonged LOS. Such an appropriate would not be applicable here because we wanted to be able to assess LOS prior to surgery. However, it does confirm the challenges of predicting longer LOSs.

There is a trade-off between precise prediction on prolonged and short LOS. For example, if we want to predict as many patients who tend to have a high LOS as possible, we can adjust the sensitivity of the classifier in our two-stage model to be higher. However, such an approach will misclassify more patients into the prolonged group and they will not receive a continuous prediction. The clinical assumption we made in the two-stage model is that the differences within the prolonged LOS group is less important from an overall resource management perspective, given the relative infrequency of prolonged LOS hospitalizations. We can adjust the model depending on the specific clinical requirements.

While our study provides some interesting insights into modeling LOS, there are some important limitations. Most importantly, one cannot conclude that the two-stage approach outlined herein will be optimal in other settings. Instead, we outline key principles for consideration when approaching this problem. It is likely that in different settings different workflows will be optimal. Concerning our own findings, we still under predict longer LOSs within 7 days, indicating that the potential for further optimization exists. Moreover, our modeling strategy, explicitly acknowledges that we cannot predict long LOS with any fidelity. Future work is needed to better model rare tail events. Additionally, we suggest, anecdotally, that that the two stage model is preferable since it is likely to engender more trust in a CDS. This is something worthy of explicit study from an implementation science perspective. Finally, while the model performs well on test data, assessment during the COVID-19 period showed worse performance, highlighting challenges of transporting models developed on pre-COVID-19 data into the COVID-19 period.

In conclusion, we have outlined different approaches for modeling a highly right skewed data like LOS. The optimal approach is driven by both empirical factors as well as the clinical use-case. We settled on a two-stage model that first classified people into long and short LOSs and then predicted actual LOS for those with a short LOS. By doing so, we make an explicit acknowledgement that we cannot predict long LOSs accurately. Doing so will hopefully engender more trust with the CDS tool. While the final model is specific to our institution and not meant to be generalizable, the modeling approach and various considerations highlight some of the complex challenges one needs to consider when developing CDS tools.

## Supplementary Information

**Additional file 1. Table S1:** Performance of the original model when classifying length of stay. **Table S2:** Variables used in the prediction model.

## Data Availability

The source data contain protected health information (PHI) and are not available for sharing. Analytic code are available upon request from Benjamin Goldstein at ben.goldstein@duke.edu.

## Abbreviations

CDS: Clinical Decision Support
CV: Cross Validation
DUHS: Duke University Health System
EHR: Electronic Health Record
LOS: Length of Stay
MAE: Mean absolute error
MLP: Multilayer perceptron
MRE: Mean relative error
MSE: Mean squared error
RF: Random Forests
