# Supplementary material for: Predicting in-hospital length of stay: a two-stage modeling approach to account for highly skewed data
Source: BMC Med Inform Decis Mak. 2022 Apr 24;22:110. doi: 10.1186/s12911-022-01855-0 (PMC9035272; doi:10.1186/s12911-022-01855-0)
Supplement: Supplementary file 1 — Additional file 1. Table S1: Performance of the original model when classifying length of stay. Table S2: Variables used in the prediction model. [file 12911_2022_1855_MOESM1_ESM.docx]

## Supplementary Information

**Table S1: Row percentages for classifications on test data in originally implemented model**

| **Actual/Predicted** | **0 – 2 Days** | **2 – 4 Days** | **4 – 7 Days** | **7+ Days** |
| --- | --- | --- | --- | --- |
| **0 – 2 Days** | 67% | 16% | 8% | 9% |
| **2 – 4 Days** | 25% | 37% | 22% | 16% |
| **4 – 7 Days** | 10% | 17% | 39% | 34% |
| **7+ Days** | 5% | 9% | 25% | 61% |

**Table S2: Predictors Used to Predict LOS**

| **VARIABLE** | **NOTES** |
| --- | --- |
| **Demographics** |  |
| Age |  |
| Sex |  |
| Race | Non-hispanic white, non-hispanic black, hispanic, other |
| Smoking Status | Ever/Never |
| BMI | Categorized as normal, overweight, obese, underweight, missing |
| **Service Utilization** | Based on past year |
| Number of previous outpatient encounters |  |
| Number of previous inpatient encounters |  |
| Number of previous emergency encounters |  |
| **Procedure Information** |  |
| CPT Code | Grouped based on CPT codes that occurred at least 75 times (~ 2x/mth) in the historic data. 150 groupings including, “other” and “none” |
| Service Line | Indicators for 12 different service lines. Defined based on service lines that appeared at least 25 times in the historic data. |
| Specialty | Indicators for 21 different specialties. Defined based on specialties that appeared at least 25 times in the historic data. |
| OR Type of Procedure | Categorized as “Major”, “Moderate”, “Minor” and “None” |
| **Comorbidities** | Based off of EPIC Groupers past 2 years |
| Diabetes |  |
| COPD |  |
| Congestive Heart Failure |  |
| Myocardial Infarction |  |
| Hypertension |  |
| Peripheral Vascular Disease |  |
| CerebroVascular Accident - Transient Ischemic Attack |  |
| Atrial Fibrillation |  |
| Atherosclerotic Cardiovascular Disease |  |
| Coronary Artery Disease |  |
| Cardiovascular Disease |  |
| Renal Diabetes |  |
| End Stage Renal Disease |  |
| Pulmonary Hypertension |  |
| Stent Placement |  |
| Cardiac Surgery |  |
| **Medication** | Based off EPIC groupers in the past 30 days |
| Hypertension Medications |  |
| ACE Inhibitors |  |
| ARBs |  |
| Beta Blockers |  |
| Calcium Channel Blockers |  |
| Digoxin |  |
| Diuretics |  |
| Nitrates |  |
| Statins |  |
| Opioids |  |
| Oral Diabetic |  |
| Anti-Coagulants |  |
| Anti-Platetlets |  |
| Anti-Arrhythmics |  |
| Psychiatric Medications |  |
| Insulin |  |
| Heart Failure Medication |  |
